# (*N*,*N*′-Di­ethyl­thio­urea-κ*S*)tris­(triphenylphosphane-κ*P*)silver(I) acetate methanol monosolvate

**DOI:** 10.1107/S1600536814010824

**Published:** 2014-05-17

**Authors:** Yupa Wattanakanjana, Arunpatcha Nimthong, Chanakan Kamrod

**Affiliations:** a Department of Chemistry, Faculty of Science, Prince of Songkla University, Hat Yai, Songkhla 90112, Thailand

## Abstract

In the mononuclear title complex, [Ag(C_5_H_12_N_2_S)(C_18_H_15_P)_3_](CH_3_COO)·CH_3_OH, the Ag^I^ ion is in a distorted tetra­hedral coordination geometry formed by three P atoms from three tri­phenyl­phosphane ligands and one S atom from an *N*,*N*′-di­ethyl­thio­urea ligand. In the crystal, the acetate anion is connected to the complex mol­ecule *via* a pair of N—H⋯O hydrogen bonds [graph-set motif *R* ^2^_2_(8)] and the solvent methanol mol­ecule is connected to the anion *via* an O—H⋯O hydrogen bond. This aggregate is further connected through a weak C—H⋯O hydrogen bond, forming a chain along [100]. In addition, sixfold phenyl embraces with inter­molecular distances of 6.6463 (13)–6.667 (2) Å are arranged in a chain along [001]. The combination of hydrogen bonding and phen­yl⋯phenyl inter­actions leads to the formation of a two-dimensional network parallel to (010).

## Related literature   

For structural reports on silver(I) complexes containing thio­urea derivatives as ligands or mixed-ligands with tri­phenyl­phosphane, see: Bowmaker *et al.* (2010 ▶); Rüffer *et al.* (2011 ▶); Pakawatchai *et al.* (2012 ▶). For potential applications of silver(I) complexes, see: Ferrari *et al.* (2007 ▶); Isab *et al.* (2010 ▶). For details of sixfold phenyl embraces, see: Dance & Scudder (2000 ▶); Scudder & Dance (2001 ▶). For hydrogen-bond graph-set analysis, see: Etter *et al.* (1990 ▶). 
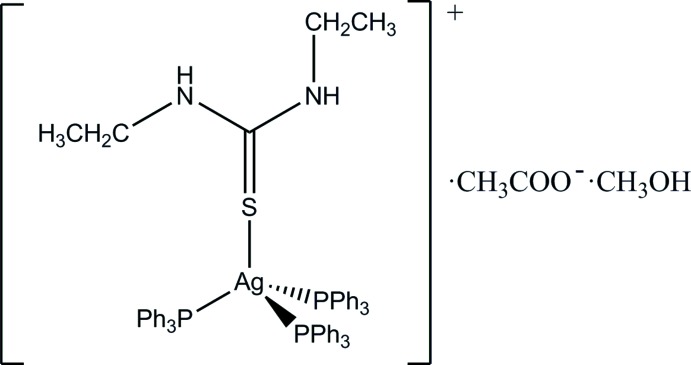


## Experimental   

### 

#### Crystal data  

[Ag(C_5_H_12_N_2_S)(C_18_H_15_P)_3_](C_2_H_3_O_2_)·CH_4_O
*M* *_r_* = 1117.99
Monoclinic, 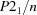

*a* = 12.950 (3) Å
*b* = 21.903 (6) Å
*c* = 19.915 (5) Å
β = 103.201 (4)°
*V* = 5500 (2) Å^3^
*Z* = 4
Mo *K*α radiation
μ = 0.54 mm^−1^
*T* = 100 K
0.18 × 0.15 × 0.11 mm

#### Data collection  

Bruker SMART APEX CCD diffractometer
Absorption correction: multi-scan (*SADABS*; Bruker, 2012 ▶) *T*_min_ = 0.608, *T*_max_ = 0.746
59032 measured reflections
16670 independent reflections
10564 reflections with *I* > 2σ(*I*)
*R*_int_ = 0.093

#### Refinement  

*R*[*F* ^2^ > 2σ(*F* ^2^)] = 0.053
*wR*(*F* ^2^) = 0.120
*S* = 1.00
16670 reflections
653 parameters
H-atom parameters constrained
Δρ_max_ = 1.23 e Å^−3^
Δρ_min_ = −1.13 e Å^−3^

### 

Data collection: *APEX2* (Bruker, 2012 ▶); cell refinement: *SAINT* (Bruker, 2012 ▶); data reduction: *SAINT*; program(s) used to solve structure: *SHELXS97* (Sheldrick, 2008 ▶); program(s) used to refine structure: *SHELXL2012* (Sheldrick, 2008 ▶) and *SHELXLE* (Hübschle *et al.*, 2011 ▶); molecular graphics: *Mercury* (Macrae *et al.*, 2008 ▶); software used to prepare material for publication: *SHELXL97* (Sheldrick, 2008 ▶) and *publCIF* (Westrip, 2010 ▶).

## Supplementary Material

Crystal structure: contains datablock(s) I. DOI: 10.1107/S1600536814010824/lh5701sup1.cif

Structure factors: contains datablock(s) I. DOI: 10.1107/S1600536814010824/lh5701Isup2.hkl

CCDC reference: 1002206

Additional supporting information:  crystallographic information; 3D view; checkCIF report

## Figures and Tables

**Table 1:** Hydrogen-bond geometry (Å, °)

*D*—H⋯*A*	*D*—H	H⋯*A*	*D*⋯*A*	*D*—H⋯*A*
N1—H1⋯O1	0.88	1.93	2.811 (3)	176
N2—H2⋯O2	0.88	1.89	2.754 (3)	168
O3—H3*D*⋯O1	0.87	1.86	2.724 (4)	177
C34—H34⋯O2^i^	0.95	2.52	3.394 (4)	154

Symmetry code: (i) 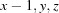.

**Fig. 1.**
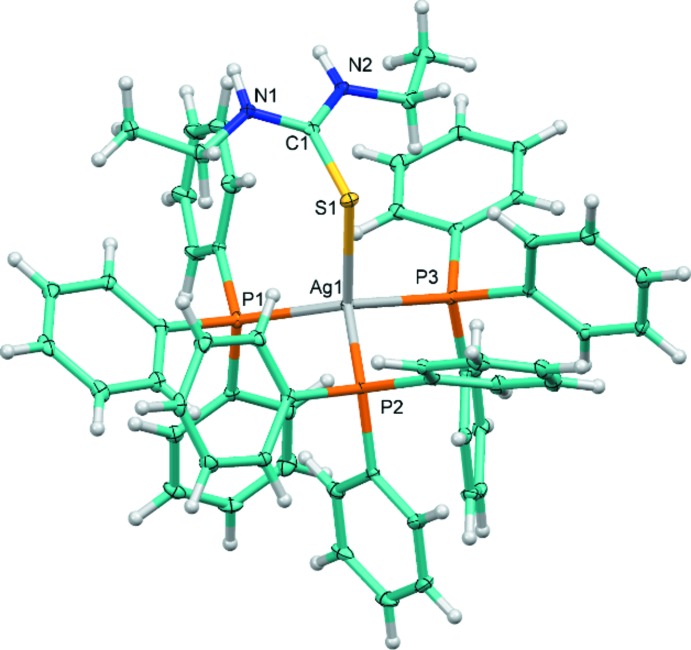
The molecular structure with displacement ellipsoids drawn at the 30% probability level. The acetate anion and methanol molecule are omitted for clarity.

**Fig. 2.**
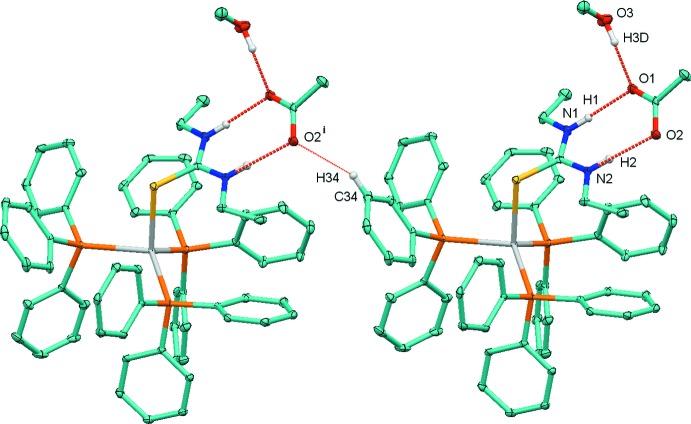
Part of the crystal structure with N—H···O and O—H···O hydrogen bonds shown as red dashed lines (symmetry code (i): x-1, y, z).

**Fig. 3.**
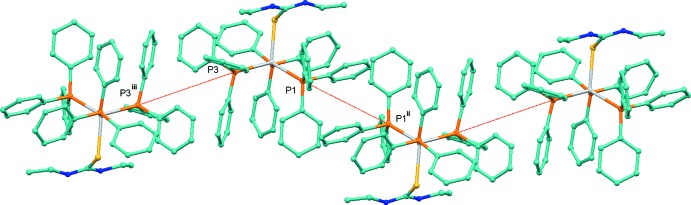
Part of the crystal structure with phenyl···phenyl interactions shown as dashed lines, which link molecules into one-dimensional zigzag chains (symmetry codes: (ii) 1-x, 1-y, -z; (iii) 1-x, 1-y, 1-z).

**Fig. 4.**
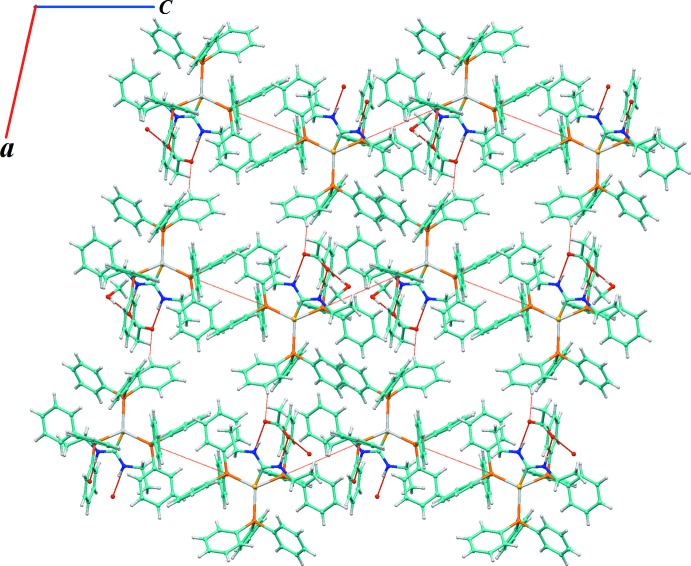
Part of the crystal structure showing formation of a layer network parallel to (010).

## supplementary crystallographic information

### 1. Experimental

Tri­phenyl­phosphane, PPh_3_ (0.31 g) was dissolved in 30 mL of methanol at 338 K and then silver acetate, AgOAc (0.10 g) was added. The mixture was stirred for 2 hr and then *N*,*N*'-di­ethyl­thio­urea, detu (0.08 g) was added and the new reaction mixture was heated under reflux 5 hr where upon the precipitate gradually disappeared. The resulting clear solution was filtered off and left to evaporate at room temperature. The crystalline complex, which deposited upon standing for several day, was filtered off and dried in vacuo.

### 1.1. Refinement

Reflections -1 1 1, 0 2 0, 0 -2 1, 1 1 0, -1 0 1, -1 -1 1, 0 0 2, 0 1 1, 0 -1 1, 0 1 2, 0 -1 2, 1 1 1, -1 3 1 and -2 2 3 were affected by the beam stop and were omitted from the refinement. All H atoms were positioned geometrically and refined using a riding-model with C—H = 0.98 Å (CH_3_), and U_iso_(H) = 1.5 U_eq_(C); 0.99 Å (CH_2_), and U_iso_(H) = 1.2 U_eq_(C); 0.95 Å (aryl H), and U_iso_(H) = 1.2 U_eq_(C); 0.88 Å (NH), and U_iso_(H) = 1.2 U_eq_(N); 0.84 Å (OH), and U_iso_(H) = 1.5 U_eq_(O).

### 2. Results and discussion

A large number of structural reports on silver(I) complexes containing thio­urea derivatives as ligands or mixed-ligands with tri­phenyl­phosphane have been studied in recent years (Bowmaker *et al.*, 2010; Rüffer *et al.*, 2011; Pakawatchai *et al.*, 2012) because these complexes have many applications. Some of these complexes show inter­esting luminescence properties (Ferrari *et al.*, 2007) and exhibit significant biological activities (Isab *et al.*, 2010). The sixfold phenyl embraces (6PE) is a common motif of a pair of threefold XPh_3_ moieties to form a concerted cycle of six edge-to-face (EF) motifs between six phenyl groups, leading to a formation one-, two-, and three-dimensional supra­molecular networks (Dance & Scudder, 2000; Scudder & Dance, 2001). Herein, we report the crystal structure a silver(I) complex containing tri­phenyl­phosphane and *N*,*N*'-di­ethyl­thio­urea.

In the mononuclear title complex, [Ag(C_5_H_12_N_2_S)(C_18_H_15_P)_3_]·CH_3_COO·CH_3_OH, the Ag^I^ ion adopts a distorted tetra­hedral geometry formed by three P atoms from three tri­phenyl­phosphane ligands, one S atom from a *N,N*'-di­ethyl­thio­urea ligand (Fig.1). The angles at the Ag^I^ ion vary from 91.66 (3)° to 115.50 (3)°. In the crystal, the acetace anion is linked to the complex molecule *via* N1—H1···O1 and N2—H2···O2 hydrogen bonds [graph-set motif R^2^_2_(8); Etter *et al.*, 1990] and the solvent methanol molecule *via* an O3—H3*D*···O1 hydrogen bond. Each cation–anion pair is linked through a weak C34(*sp*^3^)—H34···O2^i^ hydrogen bond, forming chains along the *a*-axis direction (see Table 1 and Fig. 2 ). In addition, sixfold phenyl embraces (6PE) with inter­molecular distances P1···P1^ii^ and P3···P3^iii^ of 6.6463 (13) Å and 6.667 (2) Å, respectively (symmetry code: (ii) 1-x, 1-y, -z; (iii) 1-x, 1-y, 1-z) are arranged in one-dimensional zigzag chains along the *c*-axis direction (Fig. 3). The combination of hydrogen bonding and phenyl···phenyl inter­actions lead to the formation of a layer network parallel to (010) (Fig. 4).

### Crystal data

**Table:**

[Ag(C_5_H_12_N_2_S)(C_18_H_15_P)_3_](C_2_H_3_O_2_)·CH_4_O	*F*(000) = 2328
*M**_r_* = 1117.99	*D*_x_ = 1.350 Mg m^−^^3^
Monoclinic, *P*2_1_/*n*	Mo *K*α radiation, λ = 0.71073 Å
*a* = 12.950 (3) Å	Cell parameters from 5990 reflections
*b* = 21.903 (6) Å	θ = 2.3–25.9°
*c* = 19.915 (5) Å	µ = 0.54 mm^−^^1^
β = 103.201 (4)°	*T* = 100 K
*V* = 5500 (2) Å^3^	Block, colourless
*Z* = 4	0.18 × 0.15 × 0.11 mm

### Data collection

**Table:**

Bruker SMART APEX CCD diffractometer	10564 reflections with *I* > 2σ(*I*)
Radiation source: fine focus sealed tube	*R*_int_ = 0.093
ω and π scans	θ_max_ = 30.6°, θ_min_ = 2.5°
Absorption correction: multi-scan (*SADABS*; Bruker, 2012)	*h* = −18→18
*T*_min_ = 0.608, *T*_max_ = 0.746	*k* = −30→31
59032 measured reflections	*l* = −28→27
16670 independent reflections	

### Refinement

**Table:**

Refinement on *F*^2^	Primary atom site location: structure-invariant direct methods
Least-squares matrix: full	Secondary atom site location: difference Fourier map
*R*[*F*^2^ > 2σ(*F*^2^)] = 0.053	Hydrogen site location: mixed
*wR*(*F*^2^) = 0.120	H-atom parameters constrained
*S* = 1.00	*w* = 1/[σ^2^(*F*_o_^2^) + (0.0439*P*)^2^ + 1.6753*P*] where *P* = (*F*_o_^2^ + 2*F*_c_^2^)/3
16670 reflections	(Δ/σ)_max_ = 0.002
653 parameters	Δρ_max_ = 1.23 e Å^−^^3^
0 restraints	Δρ_min_ = −1.13 e Å^−^^3^

### Special details

**Table:**

Geometry. All e.s.d.'s (except the e.s.d. in the dihedral angle between two l.s. planes) are estimated using the full covariance matrix. The cell e.s.d.'s are taken into account individually in the estimation of e.s.d.'s in distances, angles and torsion angles; correlations between e.s.d.'s in cell parameters are only used when they are defined by crystal symmetry. An approximate (isotropic) treatment of cell e.s.d.'s is used for estimating e.s.d.'s involving l.s. planes.

### Fractional atomic coordinates and isotropic or equivalent isotropic displacement parameters (Å^2^)

**Table:**

	*x*	*y*	*z*	*U*_iso_*/*U*_eq_	
Ag1	0.33154 (2)	0.60067 (2)	0.22458 (2)	0.01296 (6)	
S1	0.33683 (6)	0.72215 (3)	0.22711 (4)	0.01798 (16)	
P1	0.40233 (6)	0.55336 (4)	0.12895 (4)	0.01430 (16)	
P2	0.13005 (6)	0.60018 (4)	0.19850 (4)	0.01245 (14)	
P3	0.41323 (6)	0.56197 (3)	0.34445 (4)	0.01342 (15)	
N1	0.4563 (2)	0.77443 (12)	0.14991 (12)	0.0179 (5)	
H1	0.5131	0.7954	0.1471	0.021*	
N2	0.5416 (2)	0.75355 (11)	0.26029 (12)	0.0151 (5)	
H2	0.5981	0.7696	0.2495	0.018*	
C1	0.4519 (2)	0.75171 (13)	0.21146 (15)	0.0162 (6)	
C2	0.3736 (3)	0.76719 (16)	0.08743 (16)	0.0233 (7)	
H2A	0.3153	0.7964	0.0878	0.028*	
H2B	0.3442	0.7253	0.0854	0.028*	
C3	0.4183 (3)	0.77859 (18)	0.02503 (17)	0.0306 (8)	
H3A	0.4444	0.8207	0.0261	0.046*	
H3B	0.3627	0.7723	−0.0169	0.046*	
H3C	0.4769	0.7502	0.0253	0.046*	
C4	0.5515 (3)	0.73090 (15)	0.33009 (16)	0.0213 (7)	
H4A	0.5179	0.6902	0.3285	0.026*	
H4B	0.5144	0.7589	0.3558	0.026*	
C5	0.6670 (3)	0.72634 (16)	0.36683 (17)	0.0274 (8)	
H5A	0.6990	0.7671	0.3710	0.041*	
H5B	0.7042	0.6999	0.3404	0.041*	
H5C	0.6727	0.7091	0.4129	0.041*	
C6	0.5431 (2)	0.57060 (14)	0.13891 (15)	0.0153 (6)	
C7	0.5741 (3)	0.63004 (15)	0.15736 (18)	0.0250 (7)	
H7	0.5228	0.6590	0.1640	0.030*	
C8	0.6792 (3)	0.64759 (16)	0.16616 (18)	0.0272 (8)	
H8	0.6998	0.6885	0.1784	0.033*	
C9	0.7535 (3)	0.60516 (17)	0.15701 (18)	0.0275 (8)	
H9	0.8256	0.6168	0.1625	0.033*	
C10	0.7228 (3)	0.54571 (16)	0.13985 (19)	0.0284 (8)	
H10	0.7746	0.5163	0.1349	0.034*	
C11	0.6177 (2)	0.52847 (15)	0.12977 (17)	0.0215 (7)	
H11	0.5970	0.4878	0.1166	0.026*	
C12	0.3419 (2)	0.57843 (14)	0.04166 (15)	0.0171 (6)	
C13	0.3988 (3)	0.60774 (14)	−0.00032 (16)	0.0203 (7)	
H13	0.4723	0.6157	0.0165	0.024*	
C14	0.3486 (3)	0.62551 (15)	−0.06685 (17)	0.0257 (8)	
H14	0.3879	0.6458	−0.0950	0.031*	
C15	0.2425 (3)	0.61386 (14)	−0.09221 (17)	0.0253 (8)	
H15	0.2089	0.6254	−0.1380	0.030*	
C16	0.1846 (3)	0.58513 (15)	−0.05066 (16)	0.0228 (7)	
H16	0.1112	0.5772	−0.0678	0.027*	
C17	0.2338 (3)	0.56809 (15)	0.01571 (16)	0.0209 (7)	
H17	0.1935	0.5490	0.0441	0.025*	
C18	0.3954 (2)	0.47009 (14)	0.12207 (15)	0.0162 (6)	
C19	0.4193 (3)	0.43635 (15)	0.18297 (17)	0.0243 (7)	
H19	0.4397	0.4567	0.2261	0.029*	
C20	0.4133 (3)	0.37296 (17)	0.18082 (19)	0.0304 (8)	
H20	0.4305	0.3503	0.2225	0.036*	
C21	0.3829 (3)	0.34290 (15)	0.11903 (19)	0.0262 (8)	
H21	0.3783	0.2996	0.1179	0.031*	
C22	0.3588 (3)	0.37610 (16)	0.05847 (18)	0.0249 (7)	
H22	0.3373	0.3554	0.0156	0.030*	
C23	0.3658 (2)	0.43917 (15)	0.05958 (16)	0.0197 (7)	
H23	0.3503	0.4614	0.0175	0.024*	
C24	0.0644 (2)	0.62679 (13)	0.11257 (15)	0.0147 (6)	
C25	0.1001 (3)	0.68057 (14)	0.08859 (16)	0.0194 (7)	
H25	0.1595	0.7013	0.1159	0.023*	
C26	0.0493 (3)	0.70410 (16)	0.02476 (17)	0.0261 (8)	
H26	0.0732	0.7413	0.0091	0.031*	
C27	−0.0358 (3)	0.67363 (16)	−0.01598 (16)	0.0250 (7)	
H27	−0.0705	0.6899	−0.0595	0.030*	
C28	−0.0701 (3)	0.61942 (16)	0.00689 (17)	0.0243 (7)	
H28	−0.1275	0.5979	−0.0215	0.029*	
C29	−0.0214 (2)	0.59630 (15)	0.07107 (15)	0.0191 (6)	
H29	−0.0465	0.5595	0.0869	0.023*	
C30	0.0609 (2)	0.64181 (15)	0.25510 (15)	0.0167 (6)	
C31	0.0768 (2)	0.62282 (16)	0.32355 (16)	0.0227 (7)	
H31	0.1247	0.5905	0.3400	0.027*	
C32	0.0227 (3)	0.65100 (18)	0.36770 (18)	0.0293 (8)	
H32	0.0322	0.6373	0.4140	0.035*	
C33	−0.0451 (3)	0.69919 (17)	0.34413 (18)	0.0289 (8)	
H33	−0.0820	0.7185	0.3744	0.035*	
C34	−0.0593 (3)	0.71940 (16)	0.27687 (19)	0.0278 (8)	
H34	−0.1047	0.7531	0.2613	0.033*	
C35	−0.0071 (2)	0.69032 (15)	0.23211 (16)	0.0193 (7)	
H35	−0.0179	0.7037	0.1856	0.023*	
C36	0.0815 (2)	0.52212 (14)	0.20000 (14)	0.0161 (6)	
C37	−0.0046 (3)	0.50528 (16)	0.22687 (17)	0.0243 (7)	
H37	−0.0448	0.5355	0.2437	0.029*	
C38	−0.0314 (3)	0.44386 (18)	0.22893 (18)	0.0337 (9)	
H38	−0.0895	0.4323	0.2479	0.040*	
C39	0.0252 (3)	0.39995 (17)	0.20387 (18)	0.0350 (9)	
H39	0.0061	0.3582	0.2055	0.042*	
C40	0.1096 (3)	0.41600 (15)	0.17641 (18)	0.0282 (8)	
H40	0.1485	0.3856	0.1587	0.034*	
C41	0.1375 (3)	0.47657 (15)	0.17474 (16)	0.0218 (7)	
H41	0.1960	0.4875	0.1560	0.026*	
C42	0.3968 (2)	0.47973 (14)	0.35532 (14)	0.0148 (6)	
C43	0.2959 (2)	0.45460 (14)	0.33370 (15)	0.0186 (7)	
H43	0.2371	0.4806	0.3164	0.022*	
C44	0.2799 (3)	0.39218 (15)	0.33711 (16)	0.0218 (7)	
H44	0.2107	0.3755	0.3223	0.026*	
C45	0.3657 (3)	0.35437 (15)	0.36223 (16)	0.0217 (7)	
H45	0.3553	0.3115	0.3642	0.026*	
C46	0.4659 (3)	0.37849 (14)	0.38437 (16)	0.0199 (7)	
H46	0.5243	0.3523	0.4018	0.024*	
C47	0.4817 (2)	0.44119 (14)	0.38122 (15)	0.0173 (6)	
H47	0.5508	0.4577	0.3969	0.021*	
C48	0.5559 (2)	0.57202 (13)	0.37759 (15)	0.0149 (6)	
C49	0.6217 (2)	0.56328 (14)	0.33204 (16)	0.0199 (7)	
H49	0.5919	0.5554	0.2847	0.024*	
C50	0.7317 (3)	0.56610 (15)	0.35607 (17)	0.0230 (7)	
H50	0.7764	0.5605	0.3248	0.028*	
C51	0.7756 (3)	0.57685 (14)	0.42450 (17)	0.0215 (7)	
H51	0.8506	0.5775	0.4407	0.026*	
C52	0.7108 (2)	0.58667 (14)	0.46984 (16)	0.0201 (7)	
H52	0.7414	0.5951	0.5170	0.024*	
C53	0.6008 (2)	0.58423 (14)	0.44665 (16)	0.0179 (6)	
H53	0.5566	0.5909	0.4780	0.021*	
C54	0.3525 (2)	0.59660 (14)	0.40967 (14)	0.0146 (6)	
C55	0.3145 (2)	0.56192 (15)	0.45762 (15)	0.0183 (6)	
H55	0.3230	0.5188	0.4586	0.022*	
C56	0.2643 (2)	0.58991 (15)	0.50379 (16)	0.0216 (7)	
H56	0.2366	0.5659	0.5353	0.026*	
C57	0.2545 (2)	0.65292 (16)	0.50409 (16)	0.0229 (7)	
H57	0.2214	0.6721	0.5365	0.027*	
C58	0.2928 (3)	0.68773 (16)	0.45740 (17)	0.0232 (7)	
H58	0.2866	0.7309	0.4580	0.028*	
C59	0.3404 (2)	0.65987 (15)	0.40960 (16)	0.0201 (7)	
H59	0.3648	0.6840	0.3767	0.024*	
O1	0.63350 (18)	0.84663 (11)	0.14416 (12)	0.0267 (5)	
O2	0.72376 (18)	0.81113 (11)	0.24488 (13)	0.0306 (6)	
C60	0.7099 (2)	0.84833 (14)	0.19575 (17)	0.0189 (7)	
C61	0.7909 (3)	0.89894 (18)	0.2000 (2)	0.0354 (9)	
H61A	0.8114	0.9141	0.2475	0.053*	
H61B	0.7600	0.9324	0.1692	0.053*	
H61C	0.8536	0.8831	0.1860	0.053*	
O3	0.5306 (2)	0.92864 (13)	0.04935 (13)	0.0415 (7)	
H3D	0.5652	0.9025	0.0787	0.062*	
C62	0.4790 (3)	0.9708 (2)	0.08389 (19)	0.0386 (10)	
H62A	0.4311	0.9489	0.1071	0.058*	
H62B	0.4382	0.9996	0.0505	0.058*	
H62C	0.5319	0.9931	0.1181	0.058*	

### Atomic displacement parameters (Å^2^)

**Table:**

	*U*^11^	*U*^22^	*U*^33^	*U*^12^	*U*^13^	*U*^23^
Ag1	0.01191 (10)	0.01405 (10)	0.01316 (10)	0.00088 (10)	0.00339 (8)	0.00021 (9)
S1	0.0162 (4)	0.0138 (3)	0.0248 (4)	−0.0007 (3)	0.0066 (3)	0.0009 (3)
P1	0.0138 (4)	0.0157 (4)	0.0141 (4)	0.0027 (3)	0.0046 (3)	−0.0009 (3)
P2	0.0104 (3)	0.0126 (3)	0.0146 (3)	−0.0001 (3)	0.0034 (3)	0.0007 (3)
P3	0.0135 (4)	0.0134 (4)	0.0134 (4)	0.0008 (3)	0.0032 (3)	0.0024 (3)
N1	0.0168 (13)	0.0205 (14)	0.0166 (13)	−0.0036 (11)	0.0042 (11)	−0.0018 (10)
N2	0.0150 (12)	0.0149 (13)	0.0154 (12)	0.0001 (10)	0.0035 (10)	0.0011 (10)
C1	0.0183 (15)	0.0096 (14)	0.0212 (15)	0.0003 (12)	0.0054 (13)	−0.0032 (11)
C2	0.0190 (16)	0.0315 (19)	0.0190 (16)	0.0026 (14)	0.0033 (13)	−0.0025 (14)
C3	0.036 (2)	0.036 (2)	0.0186 (17)	−0.0033 (17)	0.0035 (16)	−0.0010 (15)
C4	0.0253 (17)	0.0188 (16)	0.0196 (16)	0.0011 (14)	0.0046 (14)	0.0052 (13)
C5	0.0296 (19)	0.0205 (17)	0.0259 (18)	−0.0039 (15)	−0.0066 (15)	0.0048 (14)
C6	0.0168 (15)	0.0174 (15)	0.0126 (14)	−0.0015 (12)	0.0050 (12)	−0.0001 (11)
C7	0.0235 (17)	0.0202 (17)	0.0336 (19)	0.0017 (14)	0.0117 (15)	−0.0071 (15)
C8	0.0240 (18)	0.0214 (18)	0.038 (2)	−0.0071 (15)	0.0107 (16)	−0.0100 (15)
C9	0.0156 (15)	0.0313 (19)	0.0356 (19)	−0.0039 (15)	0.0056 (14)	−0.0097 (16)
C10	0.0160 (16)	0.0262 (19)	0.043 (2)	0.0010 (14)	0.0067 (16)	−0.0130 (16)
C11	0.0165 (15)	0.0196 (16)	0.0278 (17)	−0.0002 (13)	0.0037 (14)	−0.0092 (13)
C12	0.0205 (16)	0.0159 (15)	0.0158 (15)	0.0043 (13)	0.0061 (13)	−0.0004 (12)
C13	0.0244 (16)	0.0164 (16)	0.0205 (15)	−0.0002 (14)	0.0063 (13)	−0.0007 (12)
C14	0.040 (2)	0.0176 (16)	0.0215 (17)	−0.0024 (15)	0.0101 (16)	0.0005 (13)
C15	0.040 (2)	0.0160 (17)	0.0172 (16)	0.0061 (15)	0.0015 (15)	−0.0012 (12)
C16	0.0214 (16)	0.0245 (18)	0.0200 (16)	0.0091 (14)	−0.0009 (14)	−0.0033 (13)
C17	0.0195 (16)	0.0222 (17)	0.0217 (16)	0.0057 (14)	0.0059 (13)	−0.0010 (13)
C18	0.0137 (14)	0.0189 (15)	0.0165 (15)	0.0021 (12)	0.0043 (12)	−0.0002 (12)
C19	0.0285 (18)	0.0238 (18)	0.0209 (17)	0.0057 (15)	0.0063 (15)	0.0003 (14)
C20	0.036 (2)	0.0263 (19)	0.030 (2)	0.0079 (17)	0.0105 (17)	0.0070 (15)
C21	0.0215 (17)	0.0135 (16)	0.043 (2)	0.0002 (14)	0.0059 (16)	0.0007 (15)
C22	0.0205 (17)	0.0219 (17)	0.0300 (19)	0.0023 (14)	0.0006 (15)	−0.0062 (14)
C23	0.0152 (15)	0.0228 (17)	0.0208 (16)	0.0016 (13)	0.0035 (13)	−0.0001 (13)
C24	0.0151 (14)	0.0135 (14)	0.0162 (14)	0.0049 (12)	0.0052 (12)	0.0020 (11)
C25	0.0223 (16)	0.0154 (15)	0.0197 (16)	0.0008 (13)	0.0029 (13)	−0.0012 (12)
C26	0.035 (2)	0.0204 (17)	0.0235 (17)	0.0030 (15)	0.0084 (15)	0.0055 (14)
C27	0.0286 (18)	0.0306 (19)	0.0145 (15)	0.0071 (15)	0.0020 (14)	0.0054 (13)
C28	0.0174 (16)	0.0328 (19)	0.0212 (17)	−0.0005 (14)	0.0012 (14)	−0.0027 (14)
C29	0.0146 (14)	0.0246 (17)	0.0186 (15)	−0.0014 (14)	0.0048 (12)	−0.0015 (13)
C30	0.0093 (13)	0.0228 (16)	0.0183 (15)	−0.0050 (12)	0.0035 (12)	−0.0039 (12)
C31	0.0135 (15)	0.0341 (19)	0.0214 (17)	−0.0004 (14)	0.0059 (13)	−0.0012 (14)
C32	0.0182 (16)	0.049 (2)	0.0214 (17)	−0.0053 (16)	0.0063 (14)	−0.0056 (16)
C33	0.0197 (17)	0.041 (2)	0.0294 (19)	−0.0050 (16)	0.0118 (15)	−0.0170 (16)
C34	0.0192 (17)	0.0244 (18)	0.039 (2)	0.0013 (14)	0.0057 (16)	−0.0106 (16)
C35	0.0171 (15)	0.0195 (16)	0.0209 (16)	−0.0027 (13)	0.0034 (13)	−0.0034 (13)
C36	0.0146 (14)	0.0187 (15)	0.0124 (14)	−0.0040 (13)	−0.0026 (12)	0.0031 (12)
C37	0.0244 (18)	0.0258 (18)	0.0227 (17)	−0.0097 (15)	0.0052 (14)	−0.0001 (14)
C38	0.039 (2)	0.037 (2)	0.0239 (18)	−0.0204 (18)	0.0051 (17)	0.0076 (16)
C39	0.052 (2)	0.0178 (17)	0.0268 (18)	−0.0132 (19)	−0.0087 (17)	0.0071 (15)
C40	0.037 (2)	0.0150 (16)	0.0255 (18)	−0.0025 (15)	−0.0084 (16)	−0.0010 (13)
C41	0.0238 (17)	0.0202 (16)	0.0185 (16)	0.0009 (14)	−0.0010 (14)	−0.0004 (13)
C42	0.0177 (15)	0.0167 (15)	0.0103 (13)	0.0005 (12)	0.0040 (12)	0.0020 (11)
C43	0.0172 (15)	0.0207 (16)	0.0153 (15)	−0.0004 (13)	−0.0012 (13)	0.0056 (12)
C44	0.0186 (15)	0.0228 (17)	0.0211 (16)	−0.0072 (14)	−0.0016 (13)	0.0016 (13)
C45	0.0279 (18)	0.0163 (16)	0.0202 (16)	−0.0005 (14)	0.0042 (14)	0.0037 (12)
C46	0.0216 (16)	0.0170 (15)	0.0210 (16)	0.0038 (13)	0.0047 (14)	0.0050 (12)
C47	0.0163 (15)	0.0174 (15)	0.0167 (15)	−0.0008 (13)	0.0009 (12)	0.0030 (12)
C48	0.0151 (14)	0.0121 (14)	0.0177 (15)	−0.0001 (12)	0.0043 (12)	0.0042 (11)
C49	0.0188 (16)	0.0195 (16)	0.0202 (16)	0.0008 (13)	0.0022 (13)	−0.0005 (13)
C50	0.0189 (16)	0.0226 (17)	0.0296 (18)	0.0019 (14)	0.0096 (15)	−0.0015 (14)
C51	0.0150 (15)	0.0165 (15)	0.0304 (18)	−0.0006 (13)	−0.0001 (14)	0.0037 (13)
C52	0.0209 (16)	0.0201 (17)	0.0156 (15)	−0.0052 (13)	−0.0032 (13)	0.0039 (12)
C53	0.0185 (15)	0.0177 (15)	0.0187 (15)	−0.0023 (12)	0.0066 (13)	0.0022 (12)
C54	0.0106 (13)	0.0202 (15)	0.0123 (13)	−0.0008 (13)	0.0012 (11)	0.0006 (12)
C55	0.0188 (15)	0.0192 (16)	0.0157 (15)	−0.0013 (13)	0.0016 (13)	0.0051 (12)
C56	0.0206 (16)	0.0286 (19)	0.0165 (15)	−0.0017 (14)	0.0060 (13)	0.0068 (13)
C57	0.0153 (15)	0.034 (2)	0.0207 (16)	0.0033 (14)	0.0070 (13)	−0.0025 (14)
C58	0.0225 (17)	0.0212 (17)	0.0273 (18)	0.0030 (14)	0.0087 (14)	−0.0017 (14)
C59	0.0195 (15)	0.0208 (16)	0.0218 (16)	−0.0028 (14)	0.0082 (13)	0.0016 (13)
O1	0.0215 (12)	0.0335 (14)	0.0244 (12)	−0.0031 (11)	0.0035 (10)	0.0092 (11)
O2	0.0216 (12)	0.0336 (14)	0.0342 (14)	−0.0056 (11)	0.0010 (11)	0.0152 (11)
C60	0.0153 (15)	0.0198 (16)	0.0252 (17)	0.0018 (13)	0.0119 (13)	−0.0011 (13)
C61	0.034 (2)	0.037 (2)	0.036 (2)	−0.0158 (19)	0.0097 (17)	0.0049 (18)
O3	0.0531 (18)	0.0419 (17)	0.0307 (15)	0.0131 (14)	0.0122 (13)	0.0079 (12)
C62	0.035 (2)	0.051 (3)	0.029 (2)	0.002 (2)	0.0062 (18)	0.0029 (18)

### Geometric parameters (Å, º)

**Table:**

Ag1—P1	2.5178 (9)	C27—C28	1.381 (5)
Ag1—P3	2.5264 (9)	C27—H27	0.9500
Ag1—P2	2.5415 (10)	C28—C29	1.386 (4)
Ag1—S1	2.6619 (10)	C28—H28	0.9500
S1—C1	1.717 (3)	C29—H29	0.9500
P1—C12	1.821 (3)	C30—C35	1.389 (4)
P1—C6	1.827 (3)	C30—C31	1.395 (4)
P1—C18	1.830 (3)	C31—C32	1.387 (4)
P2—C36	1.824 (3)	C31—H31	0.9500
P2—C24	1.824 (3)	C32—C33	1.384 (5)
P2—C30	1.834 (3)	C32—H32	0.9500
P3—C48	1.828 (3)	C33—C34	1.382 (5)
P3—C54	1.829 (3)	C33—H33	0.9500
P3—C42	1.833 (3)	C34—C35	1.391 (4)
N1—C1	1.336 (4)	C34—H34	0.9500
N1—C2	1.453 (4)	C35—H35	0.9500
N1—H1	0.8806	C36—C37	1.392 (4)
N2—C1	1.334 (4)	C36—C41	1.393 (4)
N2—C4	1.454 (4)	C37—C38	1.392 (5)
N2—H2	0.8807	C37—H37	0.9500
C2—C3	1.507 (4)	C38—C39	1.371 (6)
C2—H2A	0.9900	C38—H38	0.9500
C2—H2B	0.9900	C39—C40	1.375 (5)
C3—H3A	0.9800	C39—H39	0.9500
C3—H3B	0.9800	C40—C41	1.377 (4)
C3—H3C	0.9800	C40—H40	0.9500
C4—C5	1.510 (5)	C41—H41	0.9500
C4—H4A	0.9900	C42—C47	1.388 (4)
C4—H4B	0.9900	C42—C43	1.393 (4)
C5—H5A	0.9800	C43—C44	1.387 (4)
C5—H5B	0.9800	C43—H43	0.9500
C5—H5C	0.9800	C44—C45	1.384 (4)
C6—C11	1.378 (4)	C44—H44	0.9500
C6—C7	1.387 (4)	C45—C46	1.377 (4)
C7—C8	1.386 (5)	C45—H45	0.9500
C7—H7	0.9500	C46—C47	1.392 (4)
C8—C9	1.380 (5)	C46—H46	0.9500
C8—H8	0.9500	C47—H47	0.9500
C9—C10	1.381 (5)	C48—C53	1.391 (4)
C9—H9	0.9500	C48—C49	1.392 (4)
C10—C11	1.383 (4)	C49—C50	1.396 (4)
C10—H10	0.9500	C49—H49	0.9500
C11—H11	0.9500	C50—C51	1.372 (5)
C12—C13	1.391 (4)	C50—H50	0.9500
C12—C17	1.396 (4)	C51—C52	1.383 (4)
C13—C14	1.391 (4)	C51—H51	0.9500
C13—H13	0.9500	C52—C53	1.395 (4)
C14—C15	1.375 (5)	C52—H52	0.9500
C14—H14	0.9500	C53—H53	0.9500
C15—C16	1.388 (5)	C54—C59	1.395 (4)
C15—H15	0.9500	C54—C55	1.395 (4)
C16—C17	1.381 (4)	C55—C56	1.384 (4)
C16—H16	0.9500	C55—H55	0.9500
C17—H17	0.9500	C56—C57	1.386 (5)
C18—C23	1.392 (4)	C56—H56	0.9500
C18—C19	1.393 (4)	C57—C58	1.379 (4)
C19—C20	1.391 (5)	C57—H57	0.9500
C19—H19	0.9500	C58—C59	1.388 (4)
C20—C21	1.372 (5)	C58—H58	0.9500
C20—H20	0.9500	C59—H59	0.9500
C21—C22	1.382 (5)	O1—C60	1.254 (4)
C21—H21	0.9500	O2—C60	1.254 (4)
C22—C23	1.384 (5)	C60—C61	1.515 (5)
C22—H22	0.9500	C61—H61A	0.9800
C23—H23	0.9500	C61—H61B	0.9800
C24—C25	1.390 (4)	C61—H61C	0.9800
C24—C29	1.394 (4)	O3—C62	1.407 (5)
C25—C26	1.390 (4)	O3—H3D	0.8665
C25—H25	0.9500	C62—H62A	0.9800
C26—C27	1.382 (5)	C62—H62B	0.9800
C26—H26	0.9500	C62—H62C	0.9800
			
P1—Ag1—P3	115.50 (3)	C27—C26—C25	120.3 (3)
P1—Ag1—P2	112.20 (3)	C27—C26—H26	119.8
P3—Ag1—P2	112.37 (3)	C25—C26—H26	119.8
P1—Ag1—S1	114.42 (3)	C28—C27—C26	119.7 (3)
P3—Ag1—S1	108.30 (3)	C28—C27—H27	120.2
P2—Ag1—S1	91.65 (3)	C26—C27—H27	120.2
C1—S1—Ag1	113.15 (10)	C27—C28—C29	120.3 (3)
C12—P1—C6	104.00 (14)	C27—C28—H28	119.8
C12—P1—C18	103.08 (14)	C29—C28—H28	119.8
C6—P1—C18	104.15 (14)	C28—C29—C24	120.4 (3)
C12—P1—Ag1	116.54 (10)	C28—C29—H29	119.8
C6—P1—Ag1	111.05 (10)	C24—C29—H29	119.8
C18—P1—Ag1	116.55 (10)	C35—C30—C31	119.4 (3)
C36—P2—C24	103.07 (13)	C35—C30—P2	122.6 (2)
C36—P2—C30	103.75 (14)	C31—C30—P2	118.0 (2)
C24—P2—C30	103.25 (14)	C32—C31—C30	120.3 (3)
C36—P2—Ag1	110.00 (10)	C32—C31—H31	119.9
C24—P2—Ag1	115.31 (10)	C30—C31—H31	119.9
C30—P2—Ag1	119.65 (10)	C33—C32—C31	119.8 (3)
C48—P3—C54	104.88 (13)	C33—C32—H32	120.1
C48—P3—C42	102.17 (13)	C31—C32—H32	120.1
C54—P3—C42	104.08 (13)	C34—C33—C32	120.4 (3)
C48—P3—Ag1	118.12 (10)	C34—C33—H33	119.8
C54—P3—Ag1	112.13 (10)	C32—C33—H33	119.8
C42—P3—Ag1	113.98 (9)	C33—C34—C35	119.8 (3)
C1—N1—C2	124.6 (3)	C33—C34—H34	120.1
C1—N1—H1	117.7	C35—C34—H34	120.1
C2—N1—H1	117.7	C30—C35—C34	120.2 (3)
C1—N2—C4	124.0 (3)	C30—C35—H35	119.9
C1—N2—H2	117.9	C34—C35—H35	119.9
C4—N2—H2	118.1	C37—C36—C41	118.5 (3)
N2—C1—N1	116.4 (3)	C37—C36—P2	124.3 (3)
N2—C1—S1	121.7 (2)	C41—C36—P2	117.1 (2)
N1—C1—S1	121.8 (2)	C36—C37—C38	119.6 (3)
N1—C2—C3	109.9 (3)	C36—C37—H37	120.2
N1—C2—H2A	109.7	C38—C37—H37	120.2
C3—C2—H2A	109.7	C39—C38—C37	120.6 (3)
N1—C2—H2B	109.7	C39—C38—H38	119.7
C3—C2—H2B	109.7	C37—C38—H38	119.7
H2A—C2—H2B	108.2	C38—C39—C40	120.4 (3)
C2—C3—H3A	109.5	C38—C39—H39	119.8
C2—C3—H3B	109.5	C40—C39—H39	119.8
H3A—C3—H3B	109.5	C39—C40—C41	119.5 (4)
C2—C3—H3C	109.5	C39—C40—H40	120.3
H3A—C3—H3C	109.5	C41—C40—H40	120.3
H3B—C3—H3C	109.5	C40—C41—C36	121.4 (3)
N2—C4—C5	110.3 (3)	C40—C41—H41	119.3
N2—C4—H4A	109.6	C36—C41—H41	119.3
C5—C4—H4A	109.6	C47—C42—C43	118.8 (3)
N2—C4—H4B	109.6	C47—C42—P3	122.5 (2)
C5—C4—H4B	109.6	C43—C42—P3	118.6 (2)
H4A—C4—H4B	108.1	C44—C43—C42	120.9 (3)
C4—C5—H5A	109.5	C44—C43—H43	119.5
C4—C5—H5B	109.5	C42—C43—H43	119.5
H5A—C5—H5B	109.5	C45—C44—C43	119.5 (3)
C4—C5—H5C	109.5	C45—C44—H44	120.3
H5A—C5—H5C	109.5	C43—C44—H44	120.3
H5B—C5—H5C	109.5	C46—C45—C44	120.4 (3)
C11—C6—C7	119.6 (3)	C46—C45—H45	119.8
C11—C6—P1	124.0 (2)	C44—C45—H45	119.8
C7—C6—P1	116.4 (2)	C45—C46—C47	120.1 (3)
C8—C7—C6	120.7 (3)	C45—C46—H46	120.0
C8—C7—H7	119.7	C47—C46—H46	120.0
C6—C7—H7	119.7	C42—C47—C46	120.3 (3)
C9—C8—C7	119.4 (3)	C42—C47—H47	119.8
C9—C8—H8	120.3	C46—C47—H47	119.8
C7—C8—H8	120.3	C53—C48—C49	119.4 (3)
C8—C9—C10	119.8 (3)	C53—C48—P3	122.7 (2)
C8—C9—H9	120.1	C49—C48—P3	117.8 (2)
C10—C9—H9	120.1	C48—C49—C50	119.9 (3)
C9—C10—C11	120.7 (3)	C48—C49—H49	120.0
C9—C10—H10	119.6	C50—C49—H49	120.0
C11—C10—H10	119.6	C51—C50—C49	120.5 (3)
C6—C11—C10	119.7 (3)	C51—C50—H50	119.8
C6—C11—H11	120.2	C49—C50—H50	119.8
C10—C11—H11	120.2	C50—C51—C52	120.0 (3)
C13—C12—C17	118.5 (3)	C50—C51—H51	120.0
C13—C12—P1	122.8 (2)	C52—C51—H51	120.0
C17—C12—P1	118.7 (2)	C51—C52—C53	120.3 (3)
C12—C13—C14	120.3 (3)	C51—C52—H52	119.9
C12—C13—H13	119.8	C53—C52—H52	119.9
C14—C13—H13	119.8	C48—C53—C52	119.9 (3)
C15—C14—C13	120.5 (3)	C48—C53—H53	120.0
C15—C14—H14	119.7	C52—C53—H53	120.0
C13—C14—H14	119.7	C59—C54—C55	119.0 (3)
C14—C15—C16	119.7 (3)	C59—C54—P3	118.5 (2)
C14—C15—H15	120.1	C55—C54—P3	122.4 (2)
C16—C15—H15	120.1	C56—C55—C54	120.4 (3)
C17—C16—C15	119.9 (3)	C56—C55—H55	119.8
C17—C16—H16	120.0	C54—C55—H55	119.8
C15—C16—H16	120.0	C55—C56—C57	120.1 (3)
C16—C17—C12	121.0 (3)	C55—C56—H56	119.9
C16—C17—H17	119.5	C57—C56—H56	119.9
C12—C17—H17	119.5	C58—C57—C56	120.0 (3)
C23—C18—C19	118.8 (3)	C58—C57—H57	120.0
C23—C18—P1	123.4 (2)	C56—C57—H57	120.0
C19—C18—P1	117.8 (2)	C57—C58—C59	120.2 (3)
C20—C19—C18	120.2 (3)	C57—C58—H58	119.9
C20—C19—H19	119.9	C59—C58—H58	119.9
C18—C19—H19	119.9	C58—C59—C54	120.2 (3)
C21—C20—C19	120.6 (3)	C58—C59—H59	119.9
C21—C20—H20	119.7	C54—C59—H59	119.9
C19—C20—H20	119.7	O1—C60—O2	124.4 (3)
C20—C21—C22	119.5 (3)	O1—C60—C61	118.3 (3)
C20—C21—H21	120.3	O2—C60—C61	117.3 (3)
C22—C21—H21	120.3	C60—C61—H61A	109.5
C21—C22—C23	120.7 (3)	C60—C61—H61B	109.5
C21—C22—H22	119.6	H61A—C61—H61B	109.5
C23—C22—H22	119.6	C60—C61—H61C	109.5
C22—C23—C18	120.2 (3)	H61A—C61—H61C	109.5
C22—C23—H23	119.9	H61B—C61—H61C	109.5
C18—C23—H23	119.9	C62—O3—H3D	109.4
C25—C24—C29	119.0 (3)	O3—C62—H62A	109.5
C25—C24—P2	118.2 (2)	O3—C62—H62B	109.5
C29—C24—P2	122.7 (2)	H62A—C62—H62B	109.5
C24—C25—C26	120.2 (3)	O3—C62—H62C	109.5
C24—C25—H25	119.9	H62A—C62—H62C	109.5
C26—C25—H25	119.9	H62B—C62—H62C	109.5
			
C4—N2—C1—N1	−179.9 (3)	C36—P2—C30—C31	59.7 (3)
C4—N2—C1—S1	0.5 (4)	C24—P2—C30—C31	167.0 (2)
C2—N1—C1—N2	168.9 (3)	Ag1—P2—C30—C31	−63.3 (3)
C2—N1—C1—S1	−11.5 (4)	C35—C30—C31—C32	1.8 (5)
Ag1—S1—C1—N2	−79.1 (2)	P2—C30—C31—C32	−177.1 (3)
Ag1—S1—C1—N1	101.4 (2)	C30—C31—C32—C33	−1.7 (5)
C1—N1—C2—C3	−160.5 (3)	C31—C32—C33—C34	0.0 (5)
C1—N2—C4—C5	166.9 (3)	C32—C33—C34—C35	1.4 (5)
C12—P1—C6—C11	−97.3 (3)	C31—C30—C35—C34	−0.4 (5)
C18—P1—C6—C11	10.4 (3)	P2—C30—C35—C34	178.5 (2)
Ag1—P1—C6—C11	136.6 (2)	C33—C34—C35—C30	−1.2 (5)
C12—P1—C6—C7	83.2 (3)	C24—P2—C36—C37	−95.3 (3)
C18—P1—C6—C7	−169.1 (2)	C30—P2—C36—C37	12.1 (3)
Ag1—P1—C6—C7	−42.9 (3)	Ag1—P2—C36—C37	141.2 (2)
C11—C6—C7—C8	0.5 (5)	C24—P2—C36—C41	87.1 (2)
P1—C6—C7—C8	180.0 (3)	C30—P2—C36—C41	−165.5 (2)
C6—C7—C8—C9	−0.6 (5)	Ag1—P2—C36—C41	−36.4 (2)
C7—C8—C9—C10	−0.6 (5)	C41—C36—C37—C38	1.1 (5)
C8—C9—C10—C11	1.8 (6)	P2—C36—C37—C38	−176.4 (3)
C7—C6—C11—C10	0.7 (5)	C36—C37—C38—C39	−0.9 (5)
P1—C6—C11—C10	−178.7 (3)	C37—C38—C39—C40	0.1 (5)
C9—C10—C11—C6	−1.9 (5)	C38—C39—C40—C41	0.5 (5)
C6—P1—C12—C13	−4.2 (3)	C39—C40—C41—C36	−0.3 (5)
C18—P1—C12—C13	−112.7 (3)	C37—C36—C41—C40	−0.5 (5)
Ag1—P1—C12—C13	118.3 (2)	P2—C36—C41—C40	177.2 (2)
C6—P1—C12—C17	176.0 (2)	C48—P3—C42—C47	−1.8 (3)
C18—P1—C12—C17	67.6 (3)	C54—P3—C42—C47	−110.7 (3)
Ag1—P1—C12—C17	−61.4 (3)	Ag1—P3—C42—C47	126.8 (2)
C17—C12—C13—C14	−0.7 (5)	C48—P3—C42—C43	−178.7 (2)
P1—C12—C13—C14	179.5 (2)	C54—P3—C42—C43	72.4 (3)
C12—C13—C14—C15	−0.6 (5)	Ag1—P3—C42—C43	−50.1 (3)
C13—C14—C15—C16	1.2 (5)	C47—C42—C43—C44	−0.7 (4)
C14—C15—C16—C17	−0.4 (5)	P3—C42—C43—C44	176.3 (2)
C15—C16—C17—C12	−1.0 (5)	C42—C43—C44—C45	−0.1 (5)
C13—C12—C17—C16	1.6 (5)	C43—C44—C45—C46	0.7 (5)
P1—C12—C17—C16	−178.7 (2)	C44—C45—C46—C47	−0.4 (5)
C12—P1—C18—C23	9.3 (3)	C43—C42—C47—C46	1.0 (4)
C6—P1—C18—C23	−99.1 (3)	P3—C42—C47—C46	−175.9 (2)
Ag1—P1—C18—C23	138.2 (2)	C45—C46—C47—C42	−0.5 (5)
C12—P1—C18—C19	−169.4 (2)	C54—P3—C48—C53	18.9 (3)
C6—P1—C18—C19	82.2 (3)	C42—P3—C48—C53	−89.5 (3)
Ag1—P1—C18—C19	−40.5 (3)	Ag1—P3—C48—C53	144.6 (2)
C23—C18—C19—C20	0.0 (5)	C54—P3—C48—C49	−165.1 (2)
P1—C18—C19—C20	178.7 (3)	C42—P3—C48—C49	86.6 (3)
C18—C19—C20—C21	−0.7 (5)	Ag1—P3—C48—C49	−39.3 (3)
C19—C20—C21—C22	0.5 (5)	C53—C48—C49—C50	0.8 (5)
C20—C21—C22—C23	0.4 (5)	P3—C48—C49—C50	−175.4 (2)
C21—C22—C23—C18	−1.1 (5)	C48—C49—C50—C51	0.6 (5)
C19—C18—C23—C22	0.9 (5)	C49—C50—C51—C52	−1.8 (5)
P1—C18—C23—C22	−177.8 (2)	C50—C51—C52—C53	1.6 (5)
C36—P2—C24—C25	−167.0 (2)	C49—C48—C53—C52	−1.0 (4)
C30—P2—C24—C25	85.3 (3)	P3—C48—C53—C52	175.0 (2)
Ag1—P2—C24—C25	−47.1 (3)	C51—C52—C53—C48	−0.1 (5)
C36—P2—C24—C29	14.7 (3)	C48—P3—C54—C59	80.7 (3)
C30—P2—C24—C29	−93.1 (3)	C42—P3—C54—C59	−172.4 (2)
Ag1—P2—C24—C29	134.6 (2)	Ag1—P3—C54—C59	−48.7 (3)
C29—C24—C25—C26	1.4 (5)	C48—P3—C54—C55	−101.8 (3)
P2—C24—C25—C26	−177.0 (2)	C42—P3—C54—C55	5.2 (3)
C24—C25—C26—C27	−1.3 (5)	Ag1—P3—C54—C55	128.8 (2)
C25—C26—C27—C28	−0.2 (5)	C59—C54—C55—C56	0.8 (4)
C26—C27—C28—C29	1.6 (5)	P3—C54—C55—C56	−176.7 (2)
C27—C28—C29—C24	−1.4 (5)	C54—C55—C56—C57	−2.0 (5)
C25—C24—C29—C28	−0.1 (4)	C55—C56—C57—C58	1.3 (5)
P2—C24—C29—C28	178.3 (2)	C56—C57—C58—C59	0.6 (5)
C36—P2—C30—C35	−119.2 (3)	C57—C58—C59—C54	−1.8 (5)
C24—P2—C30—C35	−12.0 (3)	C55—C54—C59—C58	1.1 (4)
Ag1—P2—C30—C35	117.8 (2)	P3—C54—C59—C58	178.7 (2)

### Hydrogen-bond geometry (Å, º)

**Table:**

*D*—H···*A*	*D*—H	H···*A*	*D*···*A*	*D*—H···*A*
N1—H1···O1	0.88	1.93	2.811 (3)	176
N2—H2···O2	0.88	1.89	2.754 (3)	168
O3—H3*D*···O1	0.87	1.86	2.724 (4)	177
C34—H34···O2^i^	0.95	2.52	3.394 (4)	154

Symmetry code: (i) *x*−1, *y*, *z*.
